# Halogen-ion-driven polymorphs for high-performance nonlinear optical crystalline materials

**DOI:** 10.1039/d5sc10064j

**Published:** 2026-02-20

**Authors:** Yuwei Kang, Can Yang, Yunjie Wang, Qi Wu

**Affiliations:** a State Key Laboratory of New Textile Materials and Advanced Processing, Wuhan Textile University Wuhan 430200 China wuqi2011@whu.edu.cn; b State Key Laboratory of Functional Crystals and Devices, Fujian Institute of Research on the Structure of Matter, Chinese Academy of Sciences Fuzhou 350002 China

## Abstract

Noncentrosymmetric (NCS) crystalline materials are indispensable for nonlinear optical (NLO) applications, yet their rational design remains challenging due to thermodynamic preferences for centrosymmetric configurations. Herein, we demonstrate a halogen-driven strategy for obtaining NCS structures by leveraging competitive coordination between halide anions (X^−^) and stereochemically active Sn^2+^ centers. Precise halogen substitution induced the formation of two new polymorphs of [N(C_2_H_5_)_4_]SnBr_3_ (*Cc* and *Cmc*2_1_ phase) using different halide sources (*i.e.*, [N(C_2_H_5_)_4_]Cl or [N(C_2_H_5_)_4_]Br). Compared to the *Cc* phase (2 × KH_2_PO_4_), the resulting *Cmc*2_1_ phase exhibits exceptional second-harmonic generation (SHG) efficiency (5.6 × KH_2_PO_4_). This performance ranks among the highest for all reported Sn-based organic–inorganic hybrid NLO materials to date. Theoretical calculations indicate that the [SnBr_3_]^−^ unit is the primary source of the strong SHG response. This work establishes halogen-driven symmetry control as a viable strategy for achieving a dramatically enhanced SHG response, thereby providing a valuable reference for the rational design of high-performance NLO materials.

## Abstract 2

Halogen-driven polymorph selection: achieving tunable NCS crystallization and a record SHG response (5.6 × KDP) in Sn-based hybrid halides.

## Graphical abstract

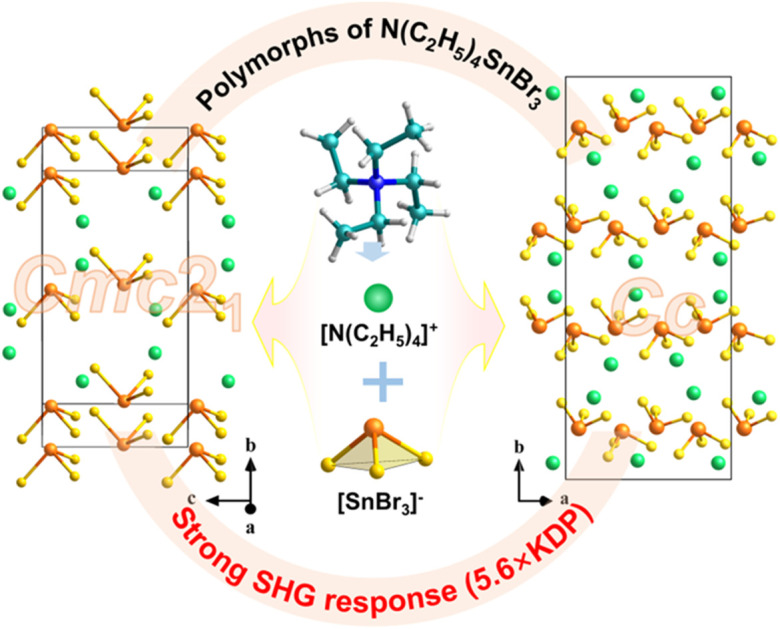

## Introduction

Nonlinear optical (NLO) crystals are pivotal for applications such as laser frequency conversion, optical communications, and quantum technologies, all of which rely on their non-centrosymmetric (NCS) structure.^1–7^ Despite this, the rational design of such crystals faces a formidable challenge: approximately 80% of all inorganic compounds crystallize in centrosymmetric (CS) structures due to the thermodynamic preference for antiparallel dipole alignment.^8–12^ Although conventional design strategies utilize asymmetric building units, such as π-conjugated anions,^13–22^ d^0^ and d^10^ transition metals,^23–28^ and lone-pair-active cations to induce structural polarity,^29–35^ achieving precise control over the formation of NCS structures remains a significant challenge.

**Fig. 1:**
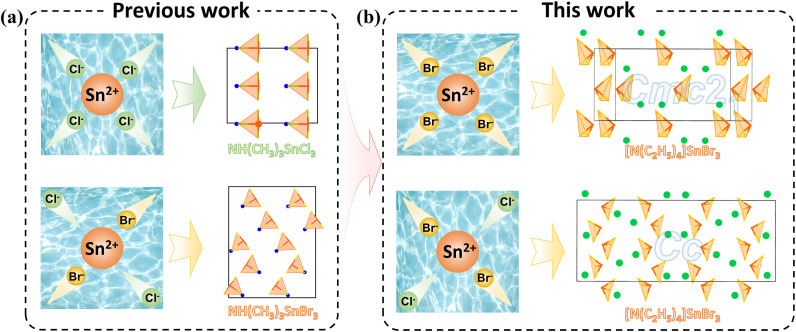
Visualization of halide-driven phase selection in NH(CH_3_)_3_SnX_3_ (X = Cl, Br) (a) and [N(C_2_H_5_)_4_]SnBr_3_ (b) based on the hard–soft acid–base principle.

By enabling diverse crystal structures under a fixed chemical composition, polymorphs significantly increase the probability of obtaining NCS compounds, thereby providing an effective route for designing high-performance NLO materials. It is this structural richness and tunable properties that make polymorphs a subject of extensive research interest.^36^ Wu *et al*. specifically reviewed NLO polymorphs and noted that, at present, most polymorphs are oxides and sulfides, obtained under different external conditions such as temperature, pressure, and the ratio of raw materials.^37^ Halides frequently exhibit polymorphism, and their synthesis, often conducted in solution, enables polymorphs' formation. Notable examples reported in recent years include α- and β-HgClBr,^38^ CsHgBr_3_,^39^ and β-RbCdI_3_·H_2_O.^40^ Tao *et al*. observed in 2016 that Sn^2+^ formed the crystal NH(CH_3_)_3_SnCl_3_ when only Cl^−^ was present in the reaction system. However, upon introducing Br^−^ into the system, Sn^2+^ preferentially coordinated with the softer Br^−^ anion to yield NH(CH_3_)_3_SnBr_3_.^41^ This behavior aligns well with the hard–soft acid–base (HSAB) principle, as the relatively soft Sn^2+^ exhibits a greater affinity for the softer halide (Br^−^ > Cl^−^) (Fig. 1a). The study clearly demonstrated the thermodynamic control exerted by the local ionic environment on product selectivity. Building on this, we further hypothesize that competitive coordination among halide ions and the central metal ion in mixed-halide systems may induce locally distorted intermediate states, which in turn can trigger the reorganization of anionic groups and ultimately drive the crystal structure toward a more thermodynamically stable phase.

Here we designed a halide-driven synthesis strategy that leverages the competitive coordination between halide anions (X^−^) and stereochemically active Sn^2+^ centers to selectively obtain distinct structural polymorphs. Specifically, using [N(C_2_H_5_)_4_]Cl as the halide source yielded a monoclinic *Cc* phase (phase II), whereas [N(C_2_H_5_)_4_]Br produced an orthorhombic polar *Cmc*2_1_ phase (phase I), demonstrating straightforward control over structural polymorphism in the [N(C_2_H_5_)_4_]SnX_3_ system. The stereochemical activity of the Sn^2+^ 5s^2^ lone-pair electrons induces pronounced distortion in the [SnX_3_]^−^ units, a feature known to favor NCS structures. Phase selectivity aligns well with the HSAB principle: as a soft acid, Sn^2+^ exhibits a stronger thermodynamic preference for the softer base Br^−^, leading to the formation of stable [SnBr_3_]^−^ units that subsequently assemble into the polar *Cmc*2_1_ phase. Notably, when Cl^−^ coexists with Br^−^ in the system, competitive coordination arises between the relatively harder base Cl^−^ and the softer Br^−^. This competition is likely to involve kinetically unstable mixed-halogen intermediates such as [SnBr_3−*x*_Cl_*x*_]^−^, which can alter the normal nucleation pathway and thereby direct crystallization toward either the lower-symmetry *Cmc*2_1_ phase or the higher-symmetry *Cc* phase (Fig. 1b). Crucially, Br^−^ owing to its greater polarizability and softer ligand character-further enhances the stereochemical activity of the Sn^2+^ lone pair within the [SnX_3_]^−^ units, driving the structural evolution from the less polar *Cc* phase to the more polar *Cmc*2_1_ phase. The highly ordered alignment of these distorted anions in the *Cmc*2_1_ phase results in a remarkable second-harmonic generation (SHG) response, the highest reported to date among Sn-based organic–inorganic hybrid NLO crystals.

This work not only reaffirms the decisive role of the HSAB principle in governing product selectivity but, more importantly, reveals a new mechanism by which halide ions kinetically steer crystallization pathways through competitive coordination. This mechanism shows that halide ions can not only dictate chemical composition through thermodynamic preference but also influence intermediate structures and crystallization kinetics, enabling the directed optimization of both crystal symmetry and macroscopic performance. The findings provide a novel rationale for the rational design of high-performance NLO crystalline materials.

## Results and discussion

The phase purity and high crystallinity of both compounds were confirmed by powder X-ray diffraction (PXRD), as shown in Fig. S1. Energy-dispersive X-ray spectroscopy (EDS) measurements yielded Sn : Br molar ratios of 1 : 3.46 and 1 : 3.01 for the respective phases. These values are in reasonable agreement with the expected stoichiometric ratio of 1 : 3 derived from single-crystal X-ray diffraction structural determinations, thus confirming the bulk composition. The crystallographic data have been deposited at the Cambridge Crystallographic Data Centre (CCDC) under deposition numbers 2518216 and 2518217.

X-ray photoelectron spectroscopy (XPS) confirms the presence of C, H, N, Sn, and Br in both compounds I and II (Fig. S2 and S3). Furthermore, the high-resolution Sn 3d spectrum exhibits a well-defined doublet with binding energies of 484.9 eV (Sn 3d_5/2_) and 493.3 eV (Sn 3d_3/2_), characterized by a spin–orbit splitting of 8.4 eV, which is a definitive signature of the Sn^2+^ oxidation state. Critically, the lack of higher binding energy features confirms the absence of detectable Sn^4+^, indicating no significant oxidation. The XPS results confirm the Sn^2+^ oxidation state.^41^ This consistency suggests that the oxidation state assigned from the structure is correct and that no significant surface oxidation has occurred, thereby supporting the overall structural assignment.

**Fig. 2:**
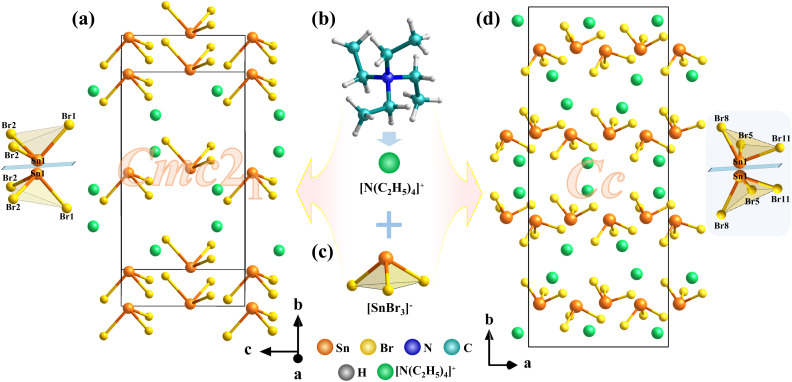
(a) Arrangement of the [SnBr_3_]^−^ in I. (b) [N(C_2_H_5_)_4_]^+^ cations. (c) [SnBr_3_]^−^ pyramid. (d) Arrangement of the [SnBr_3_]^−^ in II.

Compounds I and II crystallize in the polar space groups *Cmc*2_1_ and *Cc*, respectively. Their NCS nature is unambiguously confirmed by the observed SHG signals, despite slightly elevated Flack parameters in the structural refinements. Both compounds adopt zero-dimensional (0D) structures consisting of discrete [SnBr_3_]^−^ anions and [N(C_2_H_5_)_4_]^+^ cations (Fig. 2b and c). The [SnBr_3_]^−^ units exhibit distorted trigonal pyramidal geometries, as evidenced by Sn–Br bond lengths ranging from 2.679–2.682 Å in I and 2.653–2.710 Å in II, and Br–Sn–Br bond angles varying between 91.67–93.73° in I and 90.36–94.90° in II. These structural distortions play a crucial role in breaking symmetry. Although the two compounds share the same chemical composition, they exhibit markedly different crystal packing arrangements, which directly account for their distinct space group symmetries. In I, the [SnBr_3_]^−^ units are aligned in a highly ordered fashion along the *b*-axis, forming a mirror-like arrangement (Fig. 2a and d). In the crystal structures of both compounds, the dipole moments of the [SnBr_3_]^−^ units are arranged antiparallel along the *b*-axis, leading to mutual cancellation, while their polarities align cooperatively along the *a* and *c*-axes. This alignment results in the establishment of macroscopic non-centrosymmetry, which gives rise to a pronounced SHG response.

The infrared spectra reveal nearly identical absorption profiles for compounds I and II (Fig. S4, Table S8). Taking I as a representative example, the characteristic vibrational bands are unambiguously assigned as follows: the absorption at 2978 cm^−1^ corresponds to the asymmetric stretching vibration of C–H bonds (*ν*_as_ C–H), and the bands at 1457 cm^−1^ and 1391 cm^−1^ are attributed to the asymmetric bending vibrations of CH_3_ and CH_2_ groups (*δ*_as_ CH_3_ and *δ*_as_ CH_2_), while the features at 1183 cm^−1^ and 782 cm^−1^ are assigned to rocking modes of CH_3_ and CH_2_ moieties. All observed vibrational assignments align consistently with previously reported literature, thereby confirming the structural integrity of the organic components in the material.^42^

The optical properties of [N(C_2_H_5_)_4_]SnBr_3_ (compounds I and II) were further investigated using UV-vis absorption spectroscopy derived from the Kubelka–Munk function.^43^ As shown in Fig. S5, the optical band gaps of I and II were determined to be 3.40 eV and 3.41 eV, respectively. This mechanistic insight suggests that the fundamental electronic absorption is primarily dictated by the local [SnBr_3_]^−^ coordination geometry rather than the global crystal packing.

Differential scanning calorimetry (DSC) analysis revealed a highly reversible thermally induced solid–solid phase transition in these compounds (Fig. S6). A sharp endothermic peak was observed at 49 °C/51 °C during heating, while a symmetrical exothermic peak appeared at a similar position during cooling. The narrow thermal hysteresis indicates an extremely low energy barrier for this phase transition, confirming its excellent thermal reversibility. This low-energy characteristic suggests that the transition is likely attributable to minor structural adjustments, such as distortions within the [SnBr_3_]^−^ anion framework or conformational disordering of the organic cations, rather than a major reconstruction of the crystal lattice.

**Fig. 3:**
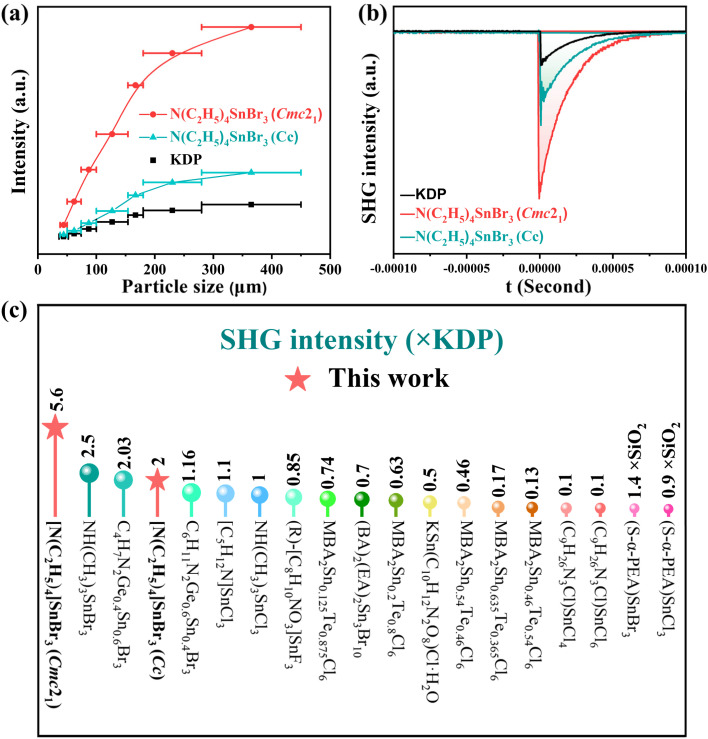
(a) SHG intensity *vs*. particle size of compounds under 1064 nm laser irradiation. (b) The oscilloscope traces of the SHG signals for powders of compounds. (c) Comparative SHG intensities among Sn-based organic–inorganic hybrid NLO crystals.

Since both compounds I and II crystallize in NCS space groups, their powder SHG effects were evaluated using the modified Kurtz–Perry method.^44^ As illustrated in Fig. 3a, under 1064 nm laser irradiation, the SHG intensities of I and II initially increase with particle size before plateauing, indicating phase-matching behavior. Quantitative measurements reveal that I and II exhibit SHG efficiencies approximately 5.6 and 2 times that of KH_2_PO_4_ (KDP), respectively (Fig. 3b). A survey of currently reported organic–inorganic hybrid Sn-based NLO crystals demonstrates that I possesses the highest SHG intensity among all such materials documented to date (Fig. 3c and Table S9).^31,41,45–51^

**Fig. 4:**
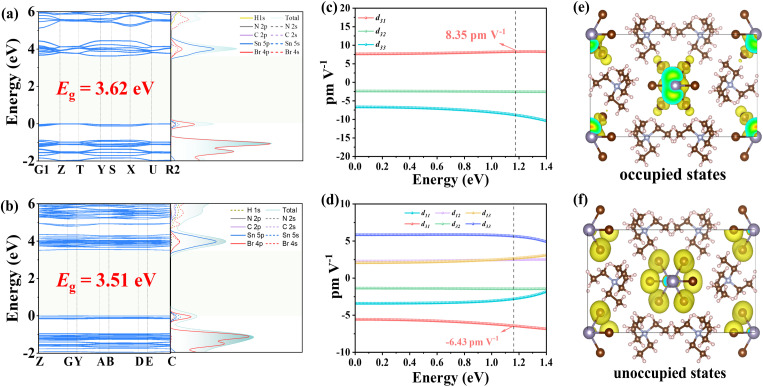
Band structure and density of states of I (a) and II (b). SHG tensor of I (c) and II (d). Occupied states (e) and unoccupied states (f) of I.

To gain deeper insights into the structure–property relationship, we performed systematic theoretical calculations. Band structure calculations reveal theoretical optical band gaps of 3.4 eV and 3.5 eV for compounds I and II, respectively, showing excellent agreement with the experimentally determined value of 3.4 eV. This close correspondence validates the reliability of our computational methodology. Density of states (DOS) analysis reveals that the valence band maximum (VBM) is dominated by contributions from the Sn-5p and Br-4s orbitals of the inorganic [SnBr_3_]^−^ units. In contrast, the conduction band minimum (CBM) is primarily constituted by the Sn-5p orbitals, with a minor contribution from Br-4s states (Fig. 4a and b). This electronic configuration demonstrates that the formation of the optical band gap is principally governed by the inorganic [SnBr_3_]^−^ trigonal pyramids. Such a well-defined electronic structure, predominantly localized on the anionic framework, provides critical insight into the origin of the SHG response, as the NLO activity can be directly correlated with these dominant transitions.

To elucidate the origin of the SHG responses in compounds I and II, we calculated their NLO coefficients (Fig. 4c and d).^52^ The results show that the largest SHG tensor component of I is *d*_31_ = 8.35 pm V^−1^, with an effective nonlinear coefficient (*d*_eff_) of 4.54 pm V^−1^. For II, the largest tensor component is *d*_31_ = −6.43 pm V^−1^, and its *d*_eff_ is 4.36 pm V^−1^. These computational results further confirm the validity of our experimental observations. Using I as an example, we further performed SHG density analysis, revealing that the primary contributions arise from the Br-4p nonbonding states (lone pairs) near the VBM and the Br antibonding orbitals at the CBM, which collectively modulate the electronic structure (Fig. 4e and f). Consequently, the pronounced SHG response of [N(C_2_H_5_)_4_]SnBr_3_ originates from the synergistic effects of the [SnBr_3_]^−^ anionic framework. Additionally, the calculated shortest phase-matching wavelengths for the two compounds are 426 nm and 476 nm, respectively (Fig. S7), indicating that phase matching is achievable at 1064 nm, which further validates the accuracy of our experimental results.

## Conclusions

In summary, this work demonstrates a halogen-mediated strategy for achieving controlled symmetry breaking and a significantly enhanced SHG response. We successfully obtained two distinct phases of [N(C_2_H_5_)_4_]SnBr_3_, the *Cc* and *Cmc*2_1_ polymorphs. The *Cmc*2_1_ phase exhibits a record SHG efficiency of 5.6 × KDP, a performance that underscores how the specific anionic arrangement is decisive in optimizing the macroscopic NLO response. Theoretical analyses confirm the [SnBr_3_]^−^ unit as the fundamental NLO-active group. Consequently, our findings establish that metal centers with stereochemically active lone pairs, when integrated with competitive coordination, provide a foundation for precise symmetry control, offering a strategic route for designing NCS materials. This approach may be generalizable to other metal-halide systems, thereby paving a promising avenue for discovering future high-performance NLO materials.

## Author contributions

Yuwei Kang: formal analysis, writing-original draft, writing-review & editing. Can Yang: methodology, data curation. Yunjie Wang: data curation, formal analysis. Qi Wu: funding acquisition, supervision, conceptualization, writing-review & editing.

## Conflicts of interest

There are no conflicts to declare.

## Supplementary Material

SC-017-D5SC10064J-s001

SC-017-D5SC10064J-s002

## Data Availability

The data that support the findings of this study are available in the supplementary information (SI) of this article. Supplementary information: experimental section and additional tables and figures. See DOI: https://doi.org/10.1039/d5sc10064j. CCDC 2518216 and 2518217 contain the supplementary crystallographic data for this paper.^53*a*,*b*^
