# Supplementary material for: Halogen-ion-driven polymorphs for high-performance nonlinear optical crystalline materials
Source: Chem Sci. 2026 Feb 20;17(16):8084–9. doi: 10.1039/d5sc10064j (PMC12947776; doi:10.1039/d5sc10064j)
Supplement: SC-017-D5SC10064J-s001 [file SC-017-D5SC10064J-s001.pdf]

Supplementary Information (SI) for Chemical Science.
This journal is © The Royal Society of Chemistry 2026

Halogen-Ion-Driven Polymorphs for High-Performance Nonlinear Optical Crystalline Materials

Yuwei Kang,[ab] Can Yang,[a] Yunjie Wang,[a] Qi Wu*[a]

[a]State Key Laboratory of New Textile Materials and Advanced Processing, Wuhan Textile University, Wuhan 430200, China

[b]State Key Laboratory of Functional Crystals and Devices, Fujian Institute of Research on the Structure of Matter, Chinese Academy of Sciences, Fuzhou 350108, China

E-mail: wuqi2011@whu.edu.cn

Experimental Procedures

1. Reagents.

Tetraethylammonium chloride (($(C_2H_5)_4NCl$, ≥99%), Tetraethylammonium bromide (($(C_2H_5)_4NBr$, ≥99%), Tetraethylammonium Iodide ($(C_2H_5)_4NI$, ≥99%), Tin (II) bromide ($SnBr_2$, ≥99%), Hydrogen bromide (HBr, 47%). All of the starting chemicals were analytical grade from a commercial source and used without further treatment.

2. Synthesis of Compounds.

Single crystal of $[N(C_2H_5)_4]SnBr_3$ (*Cmc*2_1, *Cc*) was synthesized by a simple hydrothermal method.

For $[N(C_2H_5)_4]SnBr_3$ **(*Cmc*2_1, I)** a mix of $(C_2H_5)_4NBr$ (1 mmol, 0.2795 g), $SnBr_2$ (1 mmol, 0.2785 g), and HBr (3 mL) was placed into 23 mL Teflon-lined autoclave successively. Then, the temperature was raised from room temperature to 100 °C for 2 hours and held for 1 day, slowly cooling to room temperature over 1 day. Colorless acicular crystals were obtained (75.5% base on Sn).

For $[N(C_2H_5)_4]SnBr_3$ **(*Cc*, II)**, a mix of $(C_2H_5)_4NCl$ (1 mmol, 0.1657 g), $SnBr_2$ (1 mmol, 0.2785 g), and HBr (3 mL) was placed into 23 mL Teflon-lined autoclave successively. Then, the temperature was raised from room temperature to 100 °C for 2 hours and held for 1 day, slowly cooling to room temperature over 1 day. Colorless acicular crystals were obtained (68.5% base on Sn).

3. Energy-dispersive X-ray spectroscopy (EDS).

Energy X-ray analysis was performed on $[N(C_2H_5)_4]SnBr_3$ crystal samples to obtain the elemental composition of the crystals by using energy-dispersive X-ray spectroscopy (EDS) with a field-emission scanning electron microscope (ZEISS Gemini 300).

4. Powder X-ray diffraction (XRD).

Powder X-ray diffraction (XRD) of these crystals was performed on Bruker D8 Advanced diffractometer with Cu-Kα radiation (λ= 1.54186 Å) in the angular range of 2θ =10°-70° at a scanning rate of 10° /min at room temperature.

5. Single crystal structure determination.

Single crystal X-ray diffraction data of these compounds was collected using a Bruker APEX-II CCD diffractometer equipped with a CCD detector (graphite-monochromated Cu Kα radiation, λ=1.54184 Å). Using Olex2,[1] the structure was solved with the ShelXS[2] structure solution program using Direct Methods and refined with the ShelXL[3] refinement package using Least Squares minimisation. Relevant crystallographic data and structure refinement information for the compound are summarized in Table S1-Table S4.

6. Second Harmonic Generation (SHG) Measurement.

The SHG measurements were performed on the sieved polycrystalline samples using a modified Kurtz-Perry method[4]. Polycrystalline samples of the compound were ground and sieved into several particle size ranges (35-50, 50-74, 74-100, 100-154, 154-180, 180-280 and 280-450 μm) for the measurement. Microcrystalline KH_2PO_4 (KDP) served as the standard and was sieved into the same particle size ranges and measured under the same conditions. Considering that **I** and **II** absorb moisture when left in air for a long time, the experiments of grinding into different particle sizes were carried out under infrared light and tested very quickly. The final SHG efficiency was evaluated by comparing oscilloscope traces of the SHG signals of KDP and $[N(C_2H_5)_4]SnBr_3$ **(I, II)** at the powder size of 300-400 μm.

7. UV-Vis-NIR Diffuse Reflectance Spectra.

The UV-Vis diffuse reflectance spectra were measured on a Varian Cary 2600 spectrophotometer. The $BaSO_4$ of spectral purity was used as a reference material (100 % reflectance). The absorption spectra were calculated from the reflectance spectra using the Kubelka-Munk function[5]: $\alpha/S = (1-R)^2 /2R$, where α represents the absorption coefficient, S denotes the scattering coefficient, and R is the reflectance, respectively.

8. Infrared Spectra.

IR spectra of the compound were carried out on a Nicolet iS5 Fourier-transformed infrared (FTIR) spectrometer in the range of 4000-400 cm^{-1}. The dry sample was adequately mixed with the dried KBr under the infrared light, and the sample was pressed into thin sheets under the tablet press for measurement.

9. Differential scanning calorimetry.

The differential scanning calorimetry (DSC) was performed with a Netzsch STA 449F3 analyzer.

10. Computational method.

The first-principle calculation of compounds are based on the CASTEP module of materials studio software package, and the geometric structure optimization of the system is carried out by density functional theory. The exchange correlation functional is described by the Generalized Gradient Approximation-PBE (GGA-PBE). The truncation energy of A is set to 770 eV, and the Monkhorst-Pack *k* point grid 0.04 $Å^{-1}$ is selected in the first Brillouin region to ensure the accuracy of the calculation results. Self-consistent iterative convergence (SCF) is 5.0×10^{-7} eV/atom, the maximum displacement convergence is 5.0×10^{-4} Å, the internal stress is 0.02 GPa, the force on the atom is 0.01 eV/A, and the energy convergence is within 5.0×10^{-6} eV/atom. The ion-electron interactions of all atoms are modeled by the Norm-

Conserving Pseudopotential (NCP), and the atomic electron configurations are: H $1s^1$, C $2s^22p^2$, N $2s^22p^3$, Sn $5s^25p^2$, Br $4s^24p^5$. Furthermore, the SHG coefficients are calculated using the linear norm form proposed by Aversa and Sipe.[6] After that, Rashkeev et al.[7] rearranged the equation to make the Klein-man symmetry of SHG tensor more obvious. Lin et al.[8] further rearranged Rashkeev's zero-frequency formula into contributions from virtual holes (VH), virtual electrons (VE), and two-band (TB) effects. The static second-order polarization coefficient $\chi^{(2)}$ in the zero-frequency limit can be expressed as:

$$\chi^{\alpha\beta\gamma} = \chi^{\alpha\beta\gamma}\text{ (VE)} + \chi^{\alpha\beta\gamma}\text{ (VH)} + \chi^{\alpha\beta\gamma}\text{ (two bands)}$$

where $\chi^{\alpha\beta\gamma}$ (VE) and $\chi^{\alpha\beta\gamma}$ (VH) give the contributions to $\chi^{\alpha\beta\gamma}$ from virtual-electron processes and virtual-hole processes, respectively, and $\chi^{\alpha\beta\gamma}$ (two bands) gives the contribution to $\chi^{\alpha\beta\gamma}$ from the two-band processes.

Table S1. Crystal data and structure refinement for compounds **I** and **II**.

Empirical formula	**I**	**II**
Formula weight	488.67	488.67
Temperature/K	223.00	223.0
Crystal system	orthorhombic	monoclinic
Space group	$Cmc2_1$	Cc
a/Å	10.0466	17.1273
b/Å	17.0996	33.1220
c/Å	8.7161	13.2631
α/°	90	90
β/°	90	127.506
γ/°	90	90
Volume/Å^3	1497.36	5968.7
Z	4	16
ρ_{calc} g/cm^3	2.168	2.175
μ/mm^{-1}	22.681	22.760
F (000)	920	3680
Radiation	CuK_α (λ=1.54184 Å)	CuK_α (λ=1.54178 Å)
2Θ range (°)	10.35 to 136.47 (0.83 Å)	5.34 to 136.97 (0.83 Å)
Index ranges	$-12 \leq h \leq 11$, $-17 \leq k \leq 20$, $-9 \leq l \leq 10$	$-20 \leq h \leq 18$, $-39 \leq k \leq 37$, $-15 \leq l \leq 15$
Refl. collected	5055	18370
Independent refl.	1274 [R_{int} = 0.051, R_{sigma} = 0.0477]	8509 [R_{int} = 0.1523, R_{sigma} = 0.0637]
Data/restr./param.	1274/198/108	8509/798/483
Goodness-of-fit on F^2	1.097	1.069
Final R indexes [I>2σ (I)]	R_1 = 0.0506, wR_2 = 0.1266	R_1 = 0.0839, wR_2 = 0.2198
Final R indexes [all data]	R_1 = 0.0512, wR_2 = 0.1267	R_1 = 0.0871, wR$_2$ = 0.2276
Largest diff. peak/hole / e Å^{-3}	2.68/-0.84	3.95/-2.79
Flack parameter	0.13	0.147

[a] $R_1 = \sum||Fo| - |Fc||/\sum|Fo|$, $wR_2 = \{\sum w[(Fo)^2 - (Fc)^2]^2/\sum w[(Fo)^2]^2\}^{1/2}$

Table S2. Selected fractional atomic coordinates (×10^4) and equivalent isotropic displacement parameters (Å^2×10^3) for **I**.

Atom	*x*	*y*	*z*	U_{eq}
Sn1	0.500000	0.50900(5)	0.44101(15)	0.0421(4)
Br1	0.500000	0.62801(10)	0.6414(2)	0.0589(5)
Br2	0.30540(11)	0.57526(8)	0.27585(18)	0.0576(4)

U_{eq} is defined as 1/3 of the trace of the orthogonalized U_{ij} tensor.

Table S3. Selected bond lengths (Å) for **I**.

bond	lengths (Å)
Sn1–Br1	2.682(2)
Sn1–Br2$^{\#1}$	2.6792(14)
Sn1–Br2	2.6792(14)

Table S4. Selected bond angles (°) for **I**.

bond	angles (°)
Br2–Sn1–Br1	91.67(5)
Br2$^{\#1}$–Sn1–Br1	91.67(5)
Br2–Sn1–Br2$^{\#1}$	93.73(7)

#1: 1-X, +Y, +Z;

Table S5. Selected fractional atomic coordinates (×10^4) and equivalent isotropic displacement parameters (Å^2×10^3) for **II**.

**Atom**	***x***	***y***	***z***	$\boldsymbol{U_{eq}}$
Sn1	1.05023(9)	0.63009(4)	0.45058(13)	0.0323(3)
Sn4	0.84064(9)	0.61939(4)	0.52374(12)	0.0290(3)
Sn2	0.30103(10)	0.62427(4)	0.44058(13)	0.0335(4)
Sn3	0.57885(10)	0.62663(4)	0.50534(13)	0.0338(4)
Br1	0.75607(17)	0.55655(7)	0.5510(2)	0.0361(5)
Br4	0.18433(18)	0.56277(8)	0.4012(3)	0.0447(6)
Br6	0.61024(18)	0.66255(9)	0.3499(2)	0.0467(6)
Br8	0.9659(2)	0.69272(8)	0.4833(3)	0.0531(7)
Br10	0.47581(18)	0.68986(7)	0.4915(3)	0.0448(6)
Br12	0.79463(19)	0.59101(8)	0.3063(2)	0.0435(6)
Br3	0.46440(18)	0.58791(8)	0.6418(2)	0.0448(6)
Br7	0.3222(2)	0.59280(9)	0.2707(3)	0.0509(6)
Br2	1.01792(17)	0.58203(7)	0.6641(3)	0.0465(6)
Br9	0.7438(2)	0.65742(7)	0.7128(2)	0.0470(6)
Br5	1.0428(2)	0.67076(8)	0.2684(3)	0.0518(6)
Br11	1.23363(19)	0.65400(9)	0.6410(3)	0.0521(6)

U_{eq} is defined as 1/3 of the trace of the orthogonalized U_{ij} tensor.

Table S6. Selected bond lengths (Å) for **II**.

bond	lengths (Å)	bond	lengths (Å)
Sn1–Br8	2.710(3)	Sn4–Br2	2.708(3)
Sn1–Br5	2.701(3)	Sn2–Br4	2.673(3)
Sn1–Br11	2.686(3)	Sn2–Br3	2.704(3)
Sn4–Br1	2.683(2)	Sn2–Br7	2.699(3)
Sn4–Br12	2.656(3)	Sn3–Br6	2.703(3)
Sn3–Br9	2.670(3)	Sn3–Br10	2.672(3)

Table S7. Selected bond angles (°) for **II**.

bond	lengths (Å)	bond	lengths (Å)
Br5–Sn1–Br8	91.27(10)	Br4–Sn2–Br3	93.14(9)
Br11–Sn1–Br8	93.19(10)	Br4–Sn2–Br7	90.39(9)
Br11–Sn1–Br5	94.90(10)	Br7–Sn2–Br3	93.90(9)
Br1–Sn4–Br2	91.39(8)	Br10–Sn3–Br6	91.85(9)
Br12–Sn4–Br1	92.54(8)	Br9–Sn3–Br6	92.33(10)
Br12–Sn4–Br2	92.98(9)	Br9–Sn3–Br10	90.67(9)

Table S8. Infrared Vibrations (cm^{-1}) for compounds **I** and **II**.

**I**		**II**	
Wavenumber (cm^{-1})	Assignments	Wavenumber (cm^{-1})	Assignments
2978	ν_{as}(C–H)	2980	ν_{as}(C–H)
1457	$\delta_{as}(CH_3)$	1460	$\delta_{as}(CH_3)$
1391	$\delta_{as}(CH_2)$	1390	$\delta_{as}(CH_2)$
1183	r CH_3 rocking	1180	r CH_3 rocking
999	ν_{as}(C–N)	1000	ν_{as}(C–N)
782	r CH_2 rocking	782	r CH_2 rocking

Table S9. Comparative SHG intensities among Sn-based organic-inorganic hybrid NLO crystals.

Compounds	SHG intensity (× KDP)	References
$[N(C_2H_5)_4]SnBr_3$ (Cmc2_1)	5.6	This work
$[N(C_2H_5)_4]SnBr_3$ (Cc)	2	This work
$NH(CH_3)_3SnBr_3$	2.5	9
$NH(CH_3)_3SnCl_3$	1	9
$C_4H_7N_2Ge_{0.4}Sn_{0.6}Br_3$	2.03	10
$C_6H_{11}N_2Ge_{0.6}Sn_{0.4}Br_3$	1.16	10
$[C_5H_{12}N]SnCl_3$	1.1	11
$MBA_2Sn_{0.125}Te_{0.875}Cl_6$	0.74	12
$MBA_2Sn_{0.2}Te_{0.8}Cl_6$	0.63	12
$MBA_2Sn_{0.54}Te_{0.46}Cl_6$	0.46	12
$MBA_2Sn_{0.635}Te_{0.365}Cl_6$	0.17	12
$MBA_2Sn_{0.46}Te_{0.54}Cl_6$	0.13	12
(R)-$[C_8H_{10}NO_3]SnF_3$	0.85	13
$KSn(C_{10}H_{12}N_2O_8)Cl\cdot H_2O$	0.5	14
(S-α-PEA)$SnBr_3$	1.4×α-SiO_2	15
(S-α-PEA)$SnCl_3$	0.9×α-SiO_2	15
$(C_9H_{26}N_3Cl)SnCl_4$	0.1	16
$(C_9H_{26}N_3Cl)SnCl_6$	0.1	16
$(BA)_{2(}EA)_2Sn_3Br_{10}$	0.7	17

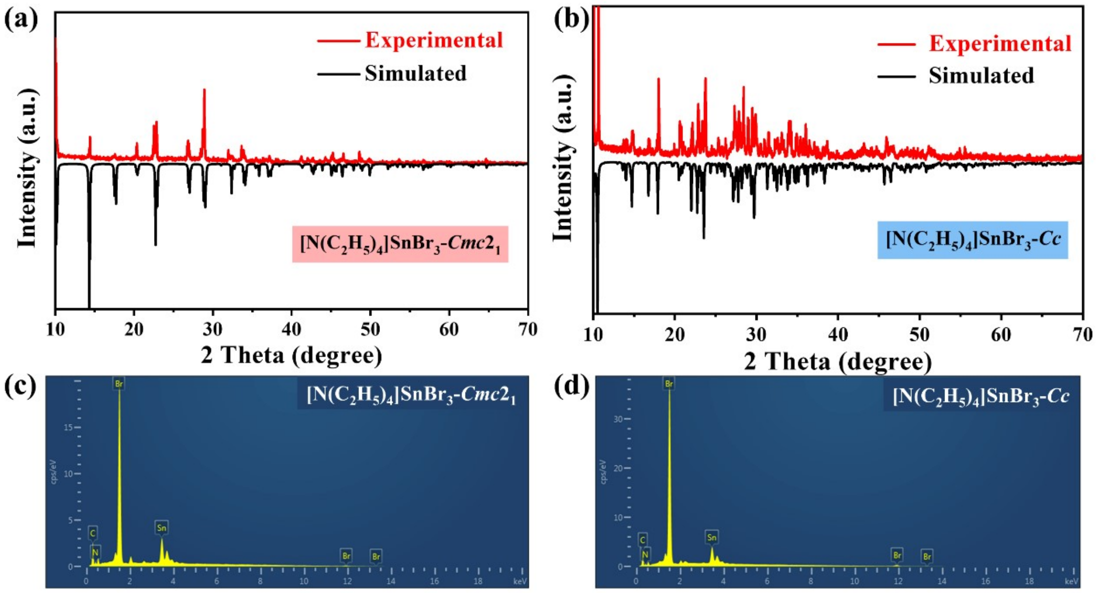

Fig. S1 XRD and EDS of compounds **I** (a) and **II** (b).

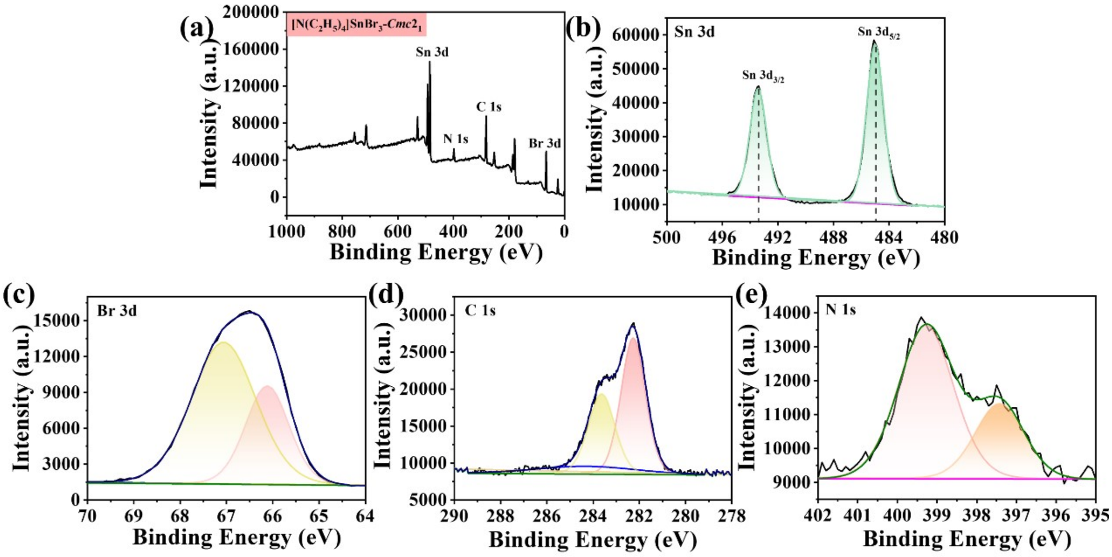

Fig. S2 XPS of $N(C_2H_5)_4SnBr_3$ (*Cmc*2_1).

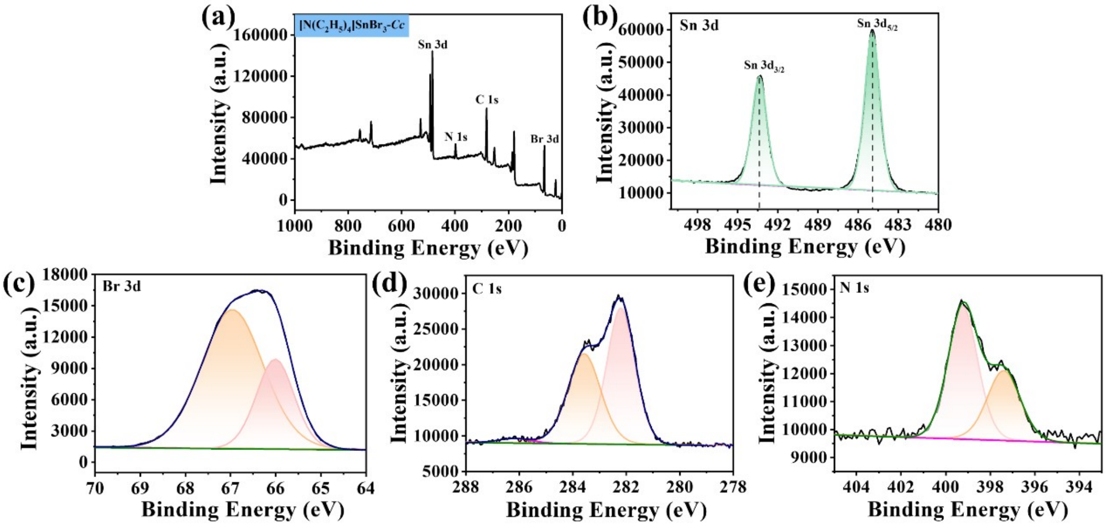

Fig. S3 XPS of $N(C_2H_5)_4SnBr_3$ (*Cc*).

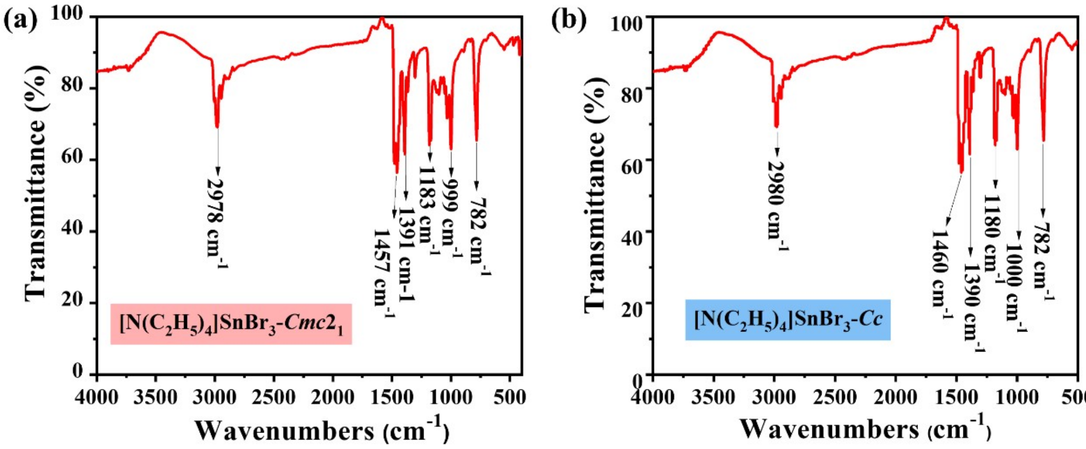

Fig. S4 The infrared spectra of compounds **I** (a) and **II** (b).

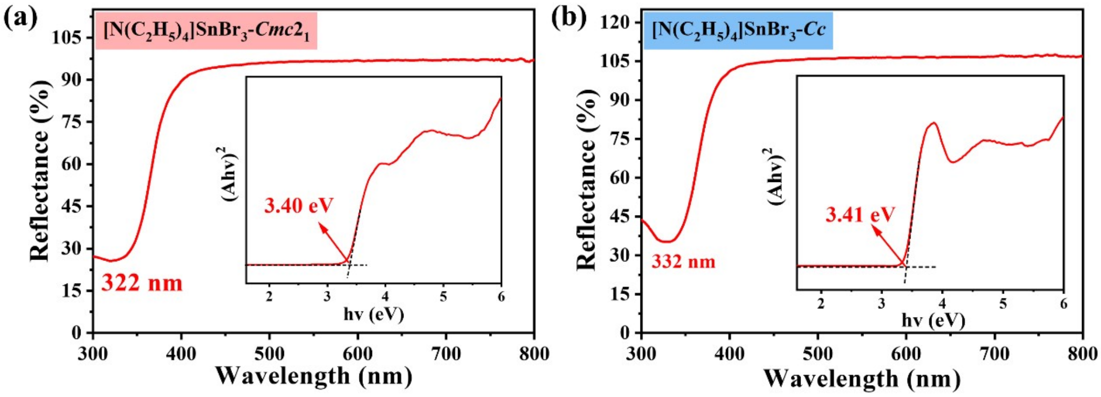

Fig. S5 The UV-Vis reflectance spectra of compounds **I** (a) and **II** (b).

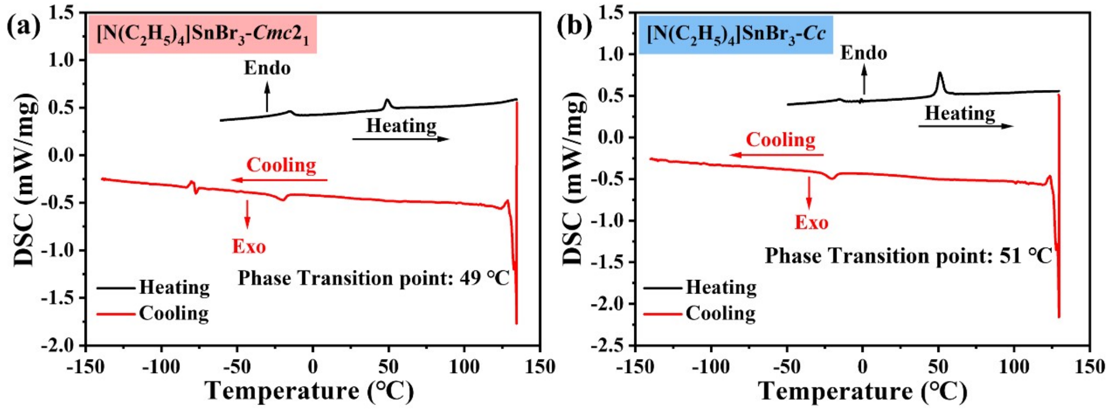

Fig. S6 DSC of compounds **I** (a) and **II** (b).

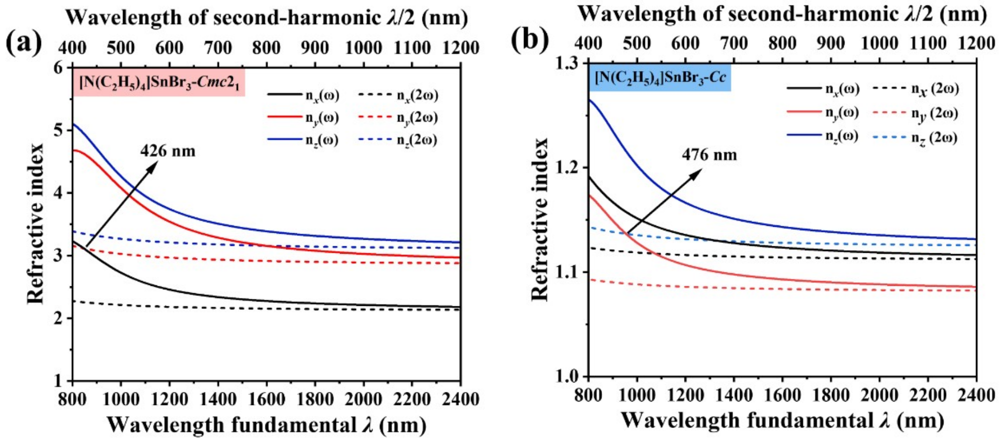

Fig. S7 Calculated refractive indices for compounds **I** (a) and **II** (b).

References

1 O. V. Dolomanov, L. J. Bourhis, R. J. Gildea, J. A. K. Howard, H. Puschmann, *J. Appl. Crystallogr.*, 2009, **42**, 339–341.

2 G. M. Sheldrick, *Acta Crystallogr. Sect. A*, 2008, **64**, 112–122.

3 G. M. Sheldrick, *Acta Crystallogr. Sect. A*, 2015, **71**, 3–8.

4 S. Kurtz, T. Perry, J. Appl. Phys. 1968, 39, 3798–3813.

5 P. Kubelka, F. Munk, Z. Tech. Phys. 1931, 12, 259–274.

6 C. Aversa and J. E. Sipe, Phys. Rev. B: Condens Matter Mater. Phys. 1995, 52, 14636.

7 S. N. Rashkeev, W. R. L. Lambrecht and B. Segall, Phys. Rev. B: Condens Matter Mater. Phys. 1998, 57, 3905.

8 J. Lin, M.-H. Lee, Z.-P. Liu, C. Chen and C. J. Pickard, Phys. Rev. B: Condens Matter Mater.Phys. 1999, **60**,13380.

9 Y. Dang, C. Zhong, G. Zhang, D. Ju, L. Wang, S. Xia, H. Xia and X. Tao, *Chem. Mater.*, 2016, **28**, 6968–6974.

10 Y. Kang, C. Yang, J. Gou, Y. Zhu, Q. Zhu and Q. Wu, *Inorg. Chem.*, 2024, **63**, 2725–2731.

11 X. Liu, C. Ji, Z. Wu, L. Li, S. Han, Y. Wang, Z. Sun and J. Luo, *Chem. Eur. J*, 2019, **25**, 2610–2615.

12 J. Wu, S.-F. Yan, W. Liu and S.-P. Guo, *Inorg. Chem.*, 2024, **63**, 17367–17371.

13 A. Jung, Y. Li and K. M. Ok, *Dalton Trans.*, 2024, **53**, 105–114.

14 Y.-H. Zhu, Z.-L. Geng, H.-X. Tang, R.-B. Fu, Z.-J. Ma and X.-T. Wu, *Scr. Mater.*, 2024, **238**, 115728.

15 K. Tao, Q. Li and Q. Yan, *Adv. Opt. Mater.*, 2024, **12**, 2400018.

16 X. Wen, G. Yi, Z. Zhang, C. Gou, H. Zeng, L. Huang, G. Zou and Z. Lin, *Dalton Trans.*, 2024, **53**, 13195–13200.

17 K. Li, X. Wang, X. Li, F. Wu, F. Zhang, Q. Wei, Z. Yue, J. Luo and X. Liu, *Inorg. Chem.*, 2024, **63**, 2275–2281.
